# DNA barcodes for delineating *Clerodendrum* species of North East India

**DOI:** 10.1038/s41598-020-70405-3

**Published:** 2020-08-10

**Authors:** Barbi Gogoi, S. B. Wann, S. P. Saikia

**Affiliations:** 1 Medicinal Aromatic and Economic Plants Group, Biological Sciences & Technology Division (BSTD), CSIR-North East Institute of Science & Technology, Jorhat, 785006 Assam India; 2 Academy of Scientific and Innovative Research (AcSIR), Ghaziabad, 201002 India; 3 Biotechnology Group, Biological Sciences & Technology Division (BSTD), CSIR-North East Institute of Science & Technology, Jorhat, 785006 Assam India

**Keywords:** Biotechnology, Molecular biology, Plant sciences

## Abstract

The diversified genus of *Clerodendrum* with its complex evolutionary history leads to taxonomic mystification. Unlike traditional taxonomic methods, DNA barcoding could be a promising tool for the identification and conservation of *Clerodendrum* species. This study was attempted to develop an efficient barcode locus in *Clerodendrum* species of North East India. We evaluated four barcode candidates (*ITS*2, *mat*K, *rbc*L, *ycf*1) and its combinations in different *Clerodendrum* species. The reliability of barcodes to distinguish the species were calculated using genetic pairwise distances, intra- and inter-specific diversity, barcode gap, and phylogenetic tree-based methods. The results exemplify that *mat*K posse’s maximum number of variables and parsimony-informative sites (103/100), intra- (0.021 ± 0.001) and inter- (0.086 ± 0.005) specific divergences and species resolution rate (89.1%) followed by *ITS*2, *ycf*1, and *rbc*L. Among the combinatorial locus, *ITS*2 + *mat*K showed the best species discrimination with distinctive barcode gaps. Therefore, we tentatively suggest that the combination of *ITS*2 + *mat*K as core barcode for *Clerodendrum* and converted into quick response (QR) code. Hence, this finding indicates that DNA barcoding could provide consistent resources for species discrimination and resolve taxonomic controversies of the genus as well as set a preliminary assessment toward its biodiversity.

## Introduction

North East India is endowed with enormous biodiversity of flora and fauna. *Clerodendrum* is a large, complex, and diversified genus that encompasses well-established pharmacological properties and its importance of ethnomedical assets was reported in many indigenous systems of medicines^1^. Globally, 540 *Clerodendrum* species were distributed in tropical and sub-tropical regions that include small trees, shrubs, and herbs^2^. Approximately, 23 *Clerodendrum* species were found in India, while 18 species occur in North East India^3^. The family of *Clerodendrum* was moved from Verbenaceae to Lamiaceae based on circumscription of evolutionary boundaries through molecular evidences^4^. Based on morphological variations, authors classified the genus into distinctive subgenera like *Clerodendrum* and *Cyclonema*, also numerous species were described by more than one authors such as *C. floribundum* Hort and *C. floribundum* R.Br., *C. foetidum* Bunge and *C. foetidum* D.Don, etc.^5,6^. Therefore, DNA barcoding techniques could function as a molecular identifier for proper documentation and classification of *Clerodendrum*. DNA barcoding uses short standardized region of DNA sequence(s) (either nuclear or/and cytoplasmic genome) for rapid authentication of discrete species and cost-effective in nature^7^. Unlike animals, the mitochondrial genes were an unsuitable choice of barcode marker in plants due to its low nucleotide substitutions rates. Subsequently, numerous nuclear and plastid genes were leading the focus of researchers for identifying plant species^8^. So far, no consensus emerged as the universal barcode for land plants^9^. However, the multi-locus combination of barcode could enhance the potential discriminatory rates between closely related species^10^. To date, no authenticated report on the practice of DNA barcoding in *Clerodendrum* sp. was cited. In this study, we collected only 9 species of *Clerodendrum* from different locations of North East India, and the rest of the species were not encountered during the fieldwork as they were extremely rare and only known from a small number of locations.

The accessibility of DNA barcoding in its practical application was constrained due to its difficulty in retrieval of information via direct scanning of DNA sequences^11^. The DNA sequences contain long strings of characters that were not practicable for data input. To resolve the issue, we attempted to develop two dimensional QR code by encoding the DNA sequences of *Clerodendrum,* which could further help any non-taxonomist to easily recognize the species in the field through direct scanning of DNA QR code label via mobile devices. Further, this study could lead to valuable aid in the conservation of biodiversity strategies and the improvement of the genus.

## Results:

### Amplification and sequencing success

The efficient PCR amplification and sequencing were regarded as a critical indicator for evaluating the barcode candidates. In this study, the success rate of PCR amplification for four loci (*ITS*2, *mat*K, *rbc*L and *ycf*1) were 100% and sequencing rates were maximum for *mat*K (95.7%) followed by *ycf*1 (94.6%), *ITS*2 (93.6%) and *rbc*L (90.4%) respectively (Table 1). A total of 352 new sequences from 9 *Clerodendrum* sp. were submitted to NCBI that includes 88, 90, 85, and 89 sequences of *ITS*2, *mat*K, *rbc*L*,* and *ycf*1. The submitted sequences were analysed together with retrieved sequences of NCBI and attained a sum of 432 sequences that consist of 118, 106, 119, and 89 sequences of *ITS*2, *mat*K, *rbc*L*,* and *ycf*1*.*

**Table 1:** Assessment of four barcodes and its combinations:

	*ITS*2	*mat*K	*rbc*L	*ycf*1	*ITS*2 + *mat*K	*ITS*2 + *rbc*L	*ITS*2 + *ycf*1	*mat*K + *rbc*L	*mat*K + *ycf*1	*rbc*L + *ycf*1	*ITS*2 + *mat*K + *rbc*L	*ITS*2 + *mat*K + *ycf*1	*ITS*2 + *rbc*L + *ycf*1	*mat*K + *rbc*L + *ycf*1	*ITS*2 + *matK* + *rbc*L + *ycf*1
No. of species samples (individuals)	118 (13)	106 (12)	119 (16)	89 (9)	102 (11)	97 (11)	88 (9)	97 (12)	87 (9)	82 (9)	93 (11)	86 (9)	82 (9)	82 (9)	80 (9)
PCR success (%)	100	100	100	100	–	–	–	–	–	–	–	–	–	–	–
Sequencing success (%)	93.6	95.7	90.4	94.6	–	–	–	–	–	–	–	–	–	–	–
Aligned sequenced length (bp)	307	759	516	872	1,067	837	1,193	1,275	1631	1,388	1583	1939	1696	2,147	2,455
No. of variable sites	98	103	20	59	264	115	139	125	158	78	219	246	162	177	164
No. of parsimony informative sites	75	100	18	52	244	104	131	119	151	75	205	228	149	169	144
Indel length	3	3	0	2	15	11	5	6	7	3	14	18	13	8	20
No. of conserved sites	209	656	496	813	865	708	1,040	1,150	1,473	1,310	1,363	1692	1533	1970	2,190

### Characteristic analysis of each barcode locus

The ambiguous terminal sequences were deleted from the aligned sequence. The length of aligned sequences for each locus and combination of locus were ranged from 307 bp of *ITS*2 to 2455 bp of *ITS*2 + *mat*K + *rbc*L + *ycf*1. Among the single locus, *mat*K had the maximum variable and parsimony-informative characters followed by *ITS*2. *ITS*2 + *mat*K had maximum variability and parsimony informative sites (264/244) among the combinational locus (Table 1). In this study, the mean inter-specific distances were much higher than intra-specific distances. The pairwise intra-specific distances among the fifteen barcodes ranged from 0.0 to 0.044 ± 0.004 and the mean intra-specific distances was maximum for *mat*K (0.021 ± 0.001) and least for *rbc*L + *ycf*1 (0.001 ± 0.000). Subsequently, the pairwise inter-specific distances were ranged from 0.0 to 0.151 ± 0.005 and the mean inter-specific distances was highest for *mat*K (0.086 ± 0.005) and least for *mat*K + *rbc*L (0.011 ± 0.003) (Table 2). In precise, *mat*K reveal the highest mean intra- and inter-specific distances.

**Table 2:** Summary of the pairwise intra-specific and inter-specific distances in *Clerodendrum* genus.

Barcode locus	Intraspecific distances (%)			Interspecific distances (%)		
	Minimum	Maximum ± S.D	Mean ± S.D	Minimum	Maximum ± S.D	Mean ± S.D
*ITS*2	0	0.029 ± 0.019	0.016 ± 0.005	0	0.100 ± 0.023	0.044 ± 0.006
*mat*K	0	0.044 ± 0.004	0.021 ± 0.001	0	0.151 ± 0.005	0.086 ± 0.005
*rbc*L	0	0.015 ± 0.005	0.008 ± 0.001	0	0.025 ± 0.006	0.019 ± 0.004
*ycf*1	0	0.018 ± 0.004	0.011 ± 0.001	0	0.032 ± 0.005	0.026 ± 0.004
*ITS*2 + *mat*K	0	0.036 ± 0.006	0.013 ± 0.002	0	0.109 ± 0.010	0.040 ± 0.006
*ITS*2 + *rbc*L	0	0.041 ± 0.007	0.007 ± 0.001	0	0.059 ± 0.008	0.027 ± 0.006
*ITS*2 + *ycf*1	0	0.037 ± 0.005	0.010 ± 0.002	0	0.058 ± 0.007	0.030 ± 0.005
*mat*K + *rbc*L	0	0.013 ± 0.002	0.002 ± 0.001	0	0.064 ± 0.007	0.011 ± 0.003
*mat*K + *ycf*1	0	0.015 ± 0.003	0.003 ± 0.001	0	0.057 ± 0.006	0.021 ± 0.003
*rbc*L + *ycf*1	0	0.013 ± 0.003	0.001 ± 0.000	0	0.025 ± 0.004	0.015 ± 0.003
*ITS*2 + *mat*K + *rbc*L	0	0.026 ± 0.004	0.006 ± 0.001	0	0.073 ± 0.007	0.018 ± 0.004
*ITS*2 + *mat*K + *ycf*1	0	0.027 ± 0.003	0.009 ± 0.001	0	0.067 ± 0.005	0.033 ± 0.003
*ITS*2 + *rbc*L + *ycf*1	0	0.028 ± 0.003	0.006 ± 0.001	0	0.045 ± 0.005	0.022 ± 0.003
*mat*K + *rbc*L + *ycf*1	0	0.013 ± 0.002	0.003 ± 0.001	0	0.046 ± 0.004	0.017 ± 0.002
*ITS*2 + *mat*K + *rbc*L + *ycf*1	0	0.022 ± 0.003	0.005 ± 0.001	0	0.054 ± 0.004	0.025 ± 0.003

*S.D* standard deviation.

### DNA barcode gap analysis

**Figure 1:**
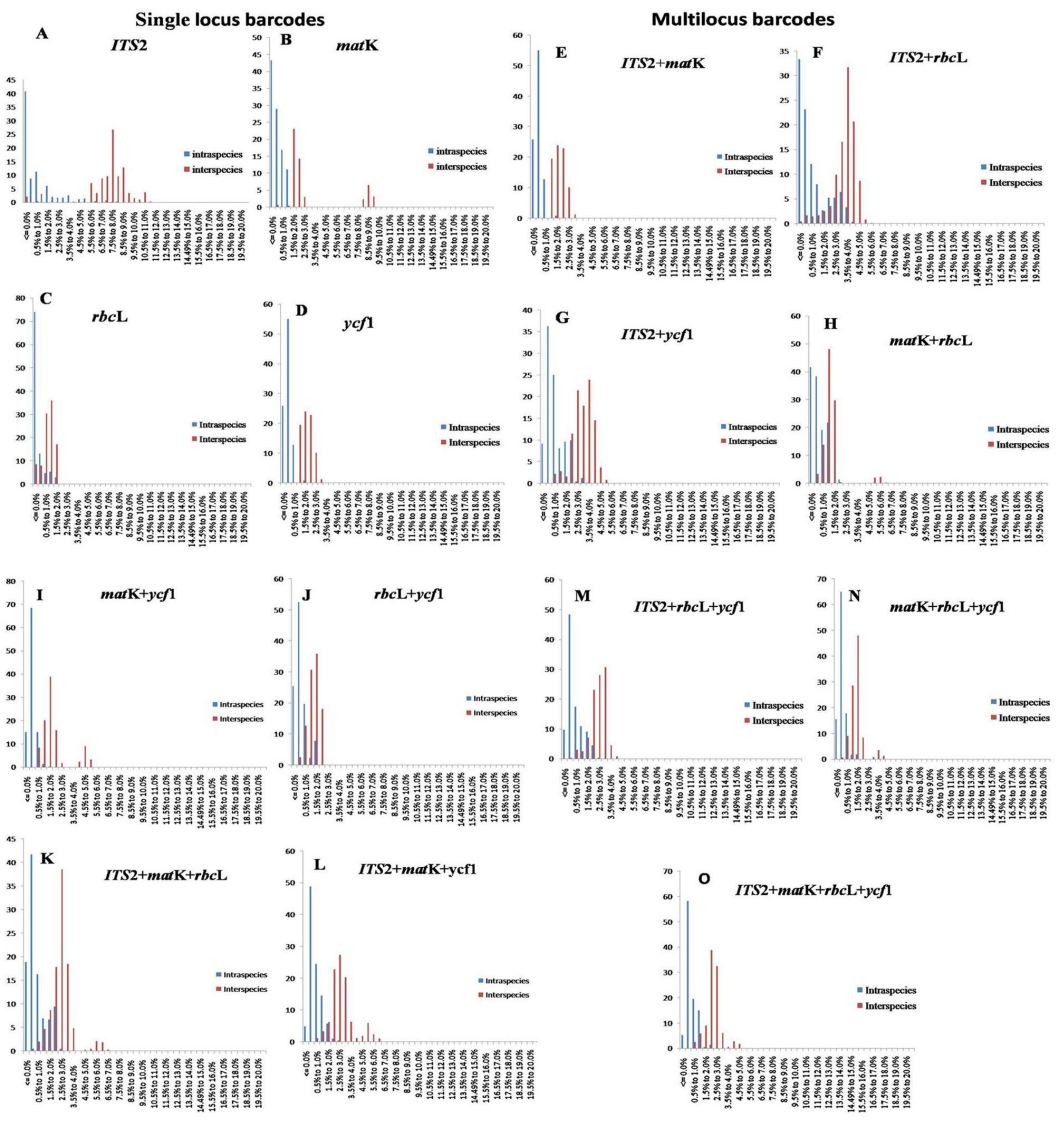
Distribution of intra- and inter-specific Kimura 2-parameter (K2P) distances among all *Clerodendrum* samples for the four barcodes loci and their combinations.

Fundamentally, an ideal barcode should show significant “barcode gap” that defined the spacer region between the range of inter and intra-specific divergences^12^. The existence of barcode gap were evaluated at a class interval of 0.005 distance units between inter and intra-specific divergences. Among the fifteen barcodes, significant barcode gap was observed in the plastid gene *mat*K, nucleotide locus *ITS*2 and *ITS*2 + *mat*K with the least overlap values, whereas the other genes revealed the unclear gaps with overlapped of intra- and inter-specific distances (Fig. 1).

### Species discrimination

For discriminating species using TaxonDNA, *ITS*2 + *mat*K had the highest success rate for correct identification of species (Best match: 96.11%; Best close match: 96.11%; All species barcodes: 84.50%) followed by *mat*K, *ITS*2, *ITS*2 + *mat*K + *ycf*1, and *rbc*L + *ycf*1 had the lowest discriminatory rate (Best match: 36.34%; Best close match: 36.34%; All species barcodes: 28.78%) (Table 3).

**Table 3:** Species identification based on the ‘best match’, ‘best close match’ and ‘all species barcodes’ with TaxonDNA software.

Regions	Best match			Best close match			All species barcodes			Threshold Value
	Correct (%)	Ambiguous (%)	Incorrect (%)	Correct (%)	Ambiguous (%)	Incorrect (%)	Correct (%)	Ambiguous (%)	Incorrect (%)	
*ITS*2	91.34	1.21	2.43	91.34	12.47	2.96	79.79	17.96	0.0	1.92
*mat*K	94.56	3.77	5.66	94.12	6.56	1.66	74.9	35.09	0.0	1.30
*rbc*L	48.48	28.15	3.36	48.48	28.15	3.36	31.09	67.22	1.68	1.35
*ycf*1	87.64	7.86	4.49	81.64	7.86	3.37	62.92	37.07	0.0	1.02
*ITS*2 + *mat*K	96.11	3.96	1.92	96.11	7.96	3.92	84.50	15.48	0.0	2.80
*ITS*2 + *rbc*L	75.25	12.37	2.37	75.25	0.0	11.25	20.61	79.38	0.0	3.30
*ITS*2 + *ycf*1	62.04	1.13	6.81	62.04	7.13	6.81	59.54	70.45	0.0	2.27
*mat*K + *rbc*L	50.72	6.18	3.09	50.32	5.06	3.09	42.26	57.73	0.0	0.86
*mat*K + *ycf*1	82.69	10.0	0.0	82.65	0.0	0.0	48.16	20.68	0.0	0.73
*rbc*L + *ycf*1	36.34	1.21	2.43	36.34	3.63	2.43	28.78	50.0	1.21	0.86
*ITS*2 + *mat*K + *rbc*L	65.69	1.07	3.22	62.04	11.07	3.22	24.73	75.26	0.0	2.01
*ITS*2 + *mat*K + *ycf*1	90.34	0.0	4.65	90.34	0.0	4.65	55.81	44.18	0.0	1.64
*ITS*2 + *rbc*L + *ycf*1	51.46	0.0	1.53	51.37	0.0	8.53	25.6	74.39	0.0	1.95
*mat*K + *rbc*L + *ycf*1	60.28	0.0	0.0	60.28	0.0	0.0	45.85	32.94	0.0	0.64
*ITS*2 + *mat*K + *rbc*L + *ycf*1	76.25	0.0	3.75	61.55	0.0	19.75	37.5	62.5	0.0	1.14

### Phylogenetic analyses

The barcode loci were analysed with BI, ML and NJ phylogenetic trees and generated similar discriminatory results with reliable clade support. The PP (Posterior Probability) values based on BI tree were higher than the bootstraps values of ML and NJ trees. The rate of discriminatory success for single and multi-locus barcodes were estimated based on percentage of species resolution for each species and determined to be monophyletic. Both the single and multi-locus barcodes showed different levels of species discrimination varying from 33.3 to 93.2% (Table 4). Amongst the single locus, *mat*K (BI-91.6, ML-91.6, NJ-91.6) followed by *ITS*2 (BI-84.6, ML-84.6, NJ-84.6) showed relatively high levels of discriminating success rates, whereas *rbc*L (BI-60.2, ML-55.2, NJ-59.6) had lowest level of discriminations. Combination of both *ITS*2 and *mat*K resolved maximum success rate of discrimination (BI-93.2, ML-91.9, NJ-93.2) as compared with other combinatorial loci of barcodes. Hence, it could be concluded that species discrimination was high when *mat*K was included among other combinations.

**Table 4:** Species discrimination rate of all barcodes loci in *Clerodendrum* species.

Regions	Species resolution (%)		
	BI	ML	NJ
*ITS*2	84.6	84.6	84.6
*mat*K	91.6	91.6	91.6
*rbc*L	60.2	55.2	59.6
*ycf*1	77.7	60.3	72.4
*ITS*2 + *mat*K	93.2	91.9	93.2
*ITS*2 + *rbc*L	63.6	52.6	54.5
*ITS*2 + *ycf*1	77.7	64.1	77.7
*mat*K + *rbc*L	59.7	58.3	59.7
*mat*K + *ycf*1	78.8	77.7	78.8
*rbc*L + *ycf*1	35.1	33.3	35.1
*ITS*2 + *mat*K + *rbc*L	72.7	63.6	72.7
*ITS*2 + *mat*K + *ycf*1	88.8	77.7	88.8
*ITS*2 + *rbc*L + *ycf*1	77.7	77.7	77.7
*mat*K + *rbc*L + *ycf*1	66.6	55.5	66.6
*ITS*2 + *mat*K + *rbc*L + *ycf*1	88.8	88.8	88.8

**Figure 2:**
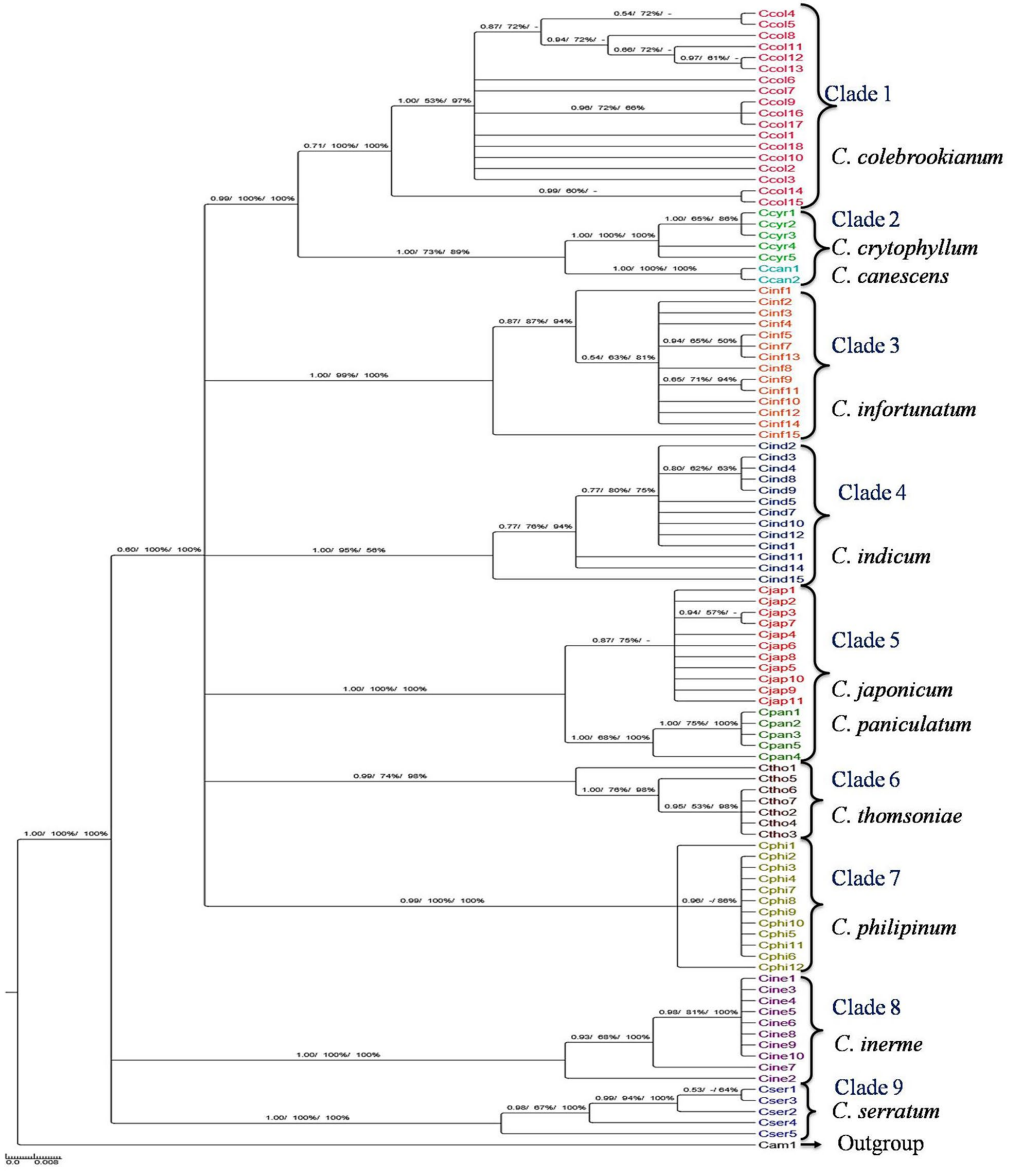
Phylogenetic BI tree inferred from ITS2 + *matK* region of *Clerodendrum *sp. Result for ML and NJ bootstrap analysis were mapped onto BI tree. The node number indicates BI/ML/NJ values. BI with PP > 0.5, ML and NJ with bootstrap > 50% were shown. The scale bar corresponds to 0.8 substitutions per 100 nucleotide positions.

The phylogenetic tree of *ITS*2 + *mat*K was reconstructed with BI method and nodal support value of ML and NJ as depicted in Fig. 2. In the phylogenetic tree, *Clerodendrum* species were well separated from outgroup and considered to be monophyletic. The phylogenetic tree was divided into 9 clades with moderate to high bootstraps and PP supports values. The Clade 1 consists of *C. colebrookianum* with 0.71 of BI support and 100% of ML and NJ bootstrap. In Clades 3, 4, 6, 7, 8, and 9 form clear individual clusters for species of *C. infortunatum*, *C. indicum*, *C. thomsoniae*, *C. philipinum*, *C. inerme,* and *C. serratum*. The species of *C. crytophyllum* and *C. canescent* in Clade 2 (BI-1.00, ML-73%, NJ-89%) and *C. japonicum* and *C. paniculatum* in Clade 5 (BI-1.00, ML-100%, NJ-100%) were grouped together in each clade which signified that they were closely related.

Thus, we tentatively proposed *ITS*2 + *mat*K gene with significant barcode gap and strong discriminatory power could be the preeminent barcode for *Clerodendrum* species.

### Two-Dimensional DNA barcode generation

**Figure 3:**
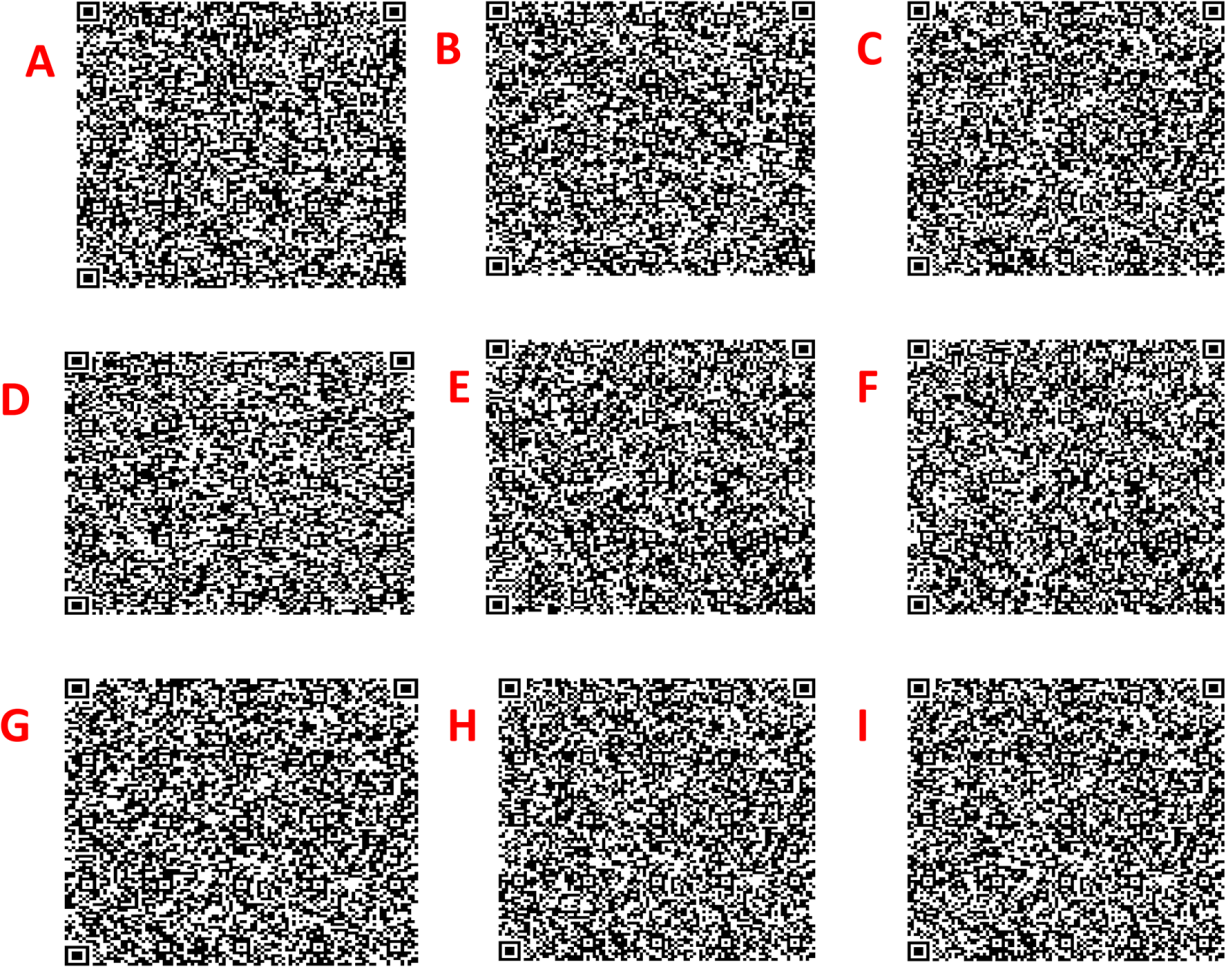
DNA sequence based QR code for species represented as (**A**) *C. colebrookianum*, (**B**) *C. infortunatum*, (**C**) *C. philipinum*, (**D**) *C. inerme*, (**E**) *C. indicum*, (**F**) *C. serratum*, (**G**) *C. thomsoniae*, (**H**) *C. paniculatum*, (**I**) *C. japonicum.*

At present, “DNA barcode” refers to the DNA sequences which were inadequate for storing data, recognition, and information retrieval. This could be resolved with the two-dimensional QR codes that could represent DNA barcode sequences efficiently. The *ITS*2 + *mat*K barcode marker of *Clerodendrum* species were transformed into QR codes with a motive to benefit the diverse researchers with no prior knowledge of DNA barcoding (Fig. 3).

## Discussion

An efficient barcode should be easily amplified, sequenced and resolve with high species discrimination and identification^13,14^. The four barcode markers used in this study were the universal plant markers with suitable length and cost effective^15^. In the present study, we analysed both the sequences of *Clerodendrum* sp. and repository sequences of GenBank records. Among the four-barcode locus, *mat*K and *ycf*1 produces high quality of sequences as compared to *ITS*2 and *rbc*L. Among the markers, *mat*K performed the best with high species resolutions and clear barcoding gaps followed by *ITS*2. The efficacy of *mat*K was also supported in previous researches as core barcode for many plants genera due to its high amount of variability and results in a high rate of molecular evolution as compared to the other barcode coding regions^16,17^. Moreover, *ITS*2 was considered as a complementary marker to the core barcodes^18,19^ and many studies had reported its high rate of variability in discriminating the species^20,21^. Many researchers proposed *ITS*2 as a standard marker for identification of more than 6,600 plant specimens from 753 genera and universal barcode for medicinal plant species^22,23^.

The plastid gene *ycf*1 was recently proposed as an effective barcode marker in angiosperms due to its high amount of variability^24^. This gene was reported to be a probable phylogenetic marker for plants like pines, orchid, etc*.*^25^. Simultaneously, the chloroplast coding region *rbc*L was proposed as a universal primer for ferns, mosses, and angiosperms^7,26^. But in some recent studies, *rbc*L was reported to be incongruous barcode marker due to its low inter-specific variations even in closely related species^20,27^. Conversely, in this study both the markers restrained the lowest number of variation site, parsimony informative sites and species discriminating rates. The complexities of these chloroplast markers prevent discrimination of species, as it represents only the maternal inheritance variation^28^. Thus, it could be suggested that *ycf*1 and *rbc*L region were not suitable for DNA barcoding in *Clerodendrum* species.

Several combinations of two, three and four barcodes were analysed in this study. The combination of *mat*K + *rbc*L was proposed to be the universal barcode for all the land plants by CBOL in 2009, but in this study, it possesses lowest species resolutions among all the combinatorial barcode markers due to its low substitution rates. In contrast, the combination of *ITS*2 + *mat*K represents the highest percentage of species resolution with clear barcode gaps as compared to both single and combination of markers and also relate similarities with the previous findings^29,30^.

The barcoding gap that exists between the highest intra-specific value and the lowest inter-specific value could depict the limits of species variation within a genus and a threshold limit of species can be set^31,32^. Overlaps of the threshold value signify cryptic species and probably show insignificant variation with the barcode. Among the single and multi-locus barcode, *mat*K and *ITS*2 + *mat*K posses’ clear barcode gap compared to the other barcode markers. The statistics of best match, best close match and all species barcodes using TaxonDNA was used to evaluate the rate of species identification^9,33^ and observed that *ITS*2 + *mat*K followed by *mat*K posses high rate of species discriminations. Based on phylogenetic tree methods, *ITS*2 + *mat*K specifies maximum rate of species resolution in *Clerodendrum*. Similar levels of resolvability by different tree-methods were reported in Lamiaceae^34^.

In recent trends, DNA barcode encounters limitation in its practical applications due to the lack of information compression and retrieval of information through direct scanning of DNA sequences^35^. Therefore, an easy innovative format and rapid retrieving barcode information is in need. Barcode technology was well established in manufacturing and retailing industries for a couple of decades. The QR code contains meaningful information in both vertical and horizontal direction more than the data carried by vertical lines of barcodes (stores maximum of 20 digits). This technique could detect symbols that lead to a specific product. If this technology was applied to represent the sequences of DNA barcodes then it could lead to efficient retrieval of information with the largest coding capacity and high compression ratio, as reported by Liu et al. In this study, *ITS*2 + *mat*K barcode sequences for 9 *Clerodendrum* sp. were converted to QR codes with vivid sequence information. The QR code could monitor the different species of *Clerodendrum* from its origin even in the field; ensure the mislabelling and safety of its commercial product.

Hence, the barcode marker *ITS*2 + *mat*K could be used as superlative locus to determine the species boundary in *Clerodendrum*. Optimization of these results for all the species of *Clerodendrum* was not advisable as this study was constraint to North East region of India but this could lay the foundation for the universal use of DNA barcoding in plants. The success rate of species identification would be more confirmed if more species were included further^36^. Therefore, a potential solution for identifying species based on geographical location and sampling size should be further investigated. In the upcoming years, these findings would be potentially helpful in delineating the large genus of *Clerodendrum*.

## Methods

### Sample collection and genomic DNA extraction

**Figure 4:**
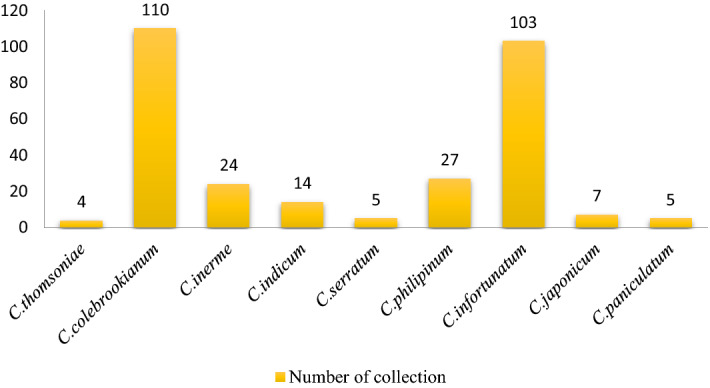
Graphical representation of collected *Clerodendrum* samples.

A total of 94 samples from 9 species of *Clerodendrum* were collected from different locations of North East India. The numbers of collected samples for each species of *Clerodendrum* were depicted in Fig. 4. Tender leaf samples were collected and lyophilized at − 110 °C for 48 h. Genomic DNA for the collected samples were extracted using modified CTAB method^37^. The quantity and quality of the extracted DNA were evaluated in Bio-spectrophotometer (Eppendorf, Germany), analysed in 0.8% agarose gel electrophoresis and visualized in gel documented system (G:BOX, Syngene, U.K.).

### PCR amplification, sequencing and sequence download

The extracted DNA samples were amplified with *ITS*2, *mat*K, *rbc*L, and *ycf*1 in polymerase chain reaction (PCR) system (Applied Biosystem). The PCR mixtures (20ul each) contained 10 ng of template DNA, 10X PCR buffer with 1.5 mM of MgCl_2_, 2.5 mM dNTP, 1 unit/uL Taq DNA polymerase, 5 pmol of each primer and adjusted the final volume with nuclease free ddH_2_O. The PCR conditions for each selected barcode primer were listed in (Table 5). For bidirectional sequencing, the amplified products were sent to Eurofins Genomics India Private Limited Company using the same primers to resolve ambiguities.

**Table 5:** Details of primer used in the study.

Regions	Primer	Sequence (5′–3′)	PCR conditions	References
ITS2	*ITS*2 F	ATGCGATACTTGGTGTGAAT	94 °C-4 min, (94 °C-30 s, 53 °C-40 s, 72 °C-40 s) 40 cycles and final extension with 72 °C-7 min	^51^
	*ITS*2 R	GACGCTTCTCCAGACTACAAT		
*matK*	3*FKIM*	CGTACAGTACTTTTGTGTTTACGAG	95 °C-4 min, (95 °C-30 s, 50 °C-40 s, 72 °C-50 s) 35 cycles and final extension with 72 °C-2 min	^52^
	1*RKIM*	ACCCAGTCCATCTGGAAATCTTGGTTC		
*rbcL*	*aF*	ATGTCACCACAAACAGAGACTAAAGC	94 °C-7 min, (94 °C-1 min, 51 °C-30 s, 72 °C-1 min) 35 cycles and final extension with 72 °C-10 min	^53^
	*aR*	GTAAAATCAAGTCCACCRCG		
*ycf*1	*F*	TCTCGACGAAAATCAGATTGTTGTGAAT	94 °C-4 min, (94 °C-30 s, 52 °C-40 s, 72 °C-1 min) 35 cycles and final extension with 72 °C-10 min	^24^
	*R*	ATACATGTCAAAGTGATGGAAA		

Additionally, we retrieved all the sequences (*ITS*2, *mat*K, *rbc*L and *ycf*1) of *Clerodendrum* from NCBI database. The downloaded sequences were filtered based on the criteria that: (i) sequence length less than 300 bp and (ii) sequences lacking specific voucher names. According to our survey, some species contain less than five sequences in NCBI while some species had maximum number of sequences for a specific barcode region. Therefore, the representatives for each species were restricted between five to eighteen samples. The taxa, voucher names and accession number used in this study were provided in (Table S1).

### Data analysis

The sequences of each barcode were aligned with MUSCLE (https://www.ebi.ac.uk/Tools/msa/muscle) and edited manually in BioEdit v7.1.3.0^38^. For *ITS*2 region, the sequences were subjected to Hidden Markov Model (HMM) to remove the conserved 5.8Sand 28S DNA sequences^39^. The edited sequences were compared with available nucleotide sequences of GenBank database and submitted to NCBI and BOLD databases with project code-NECLE (Table S2). The analyses of genetic pairwise distances were computed in MEGA X^40^ with Kimura-2-parameter (K2P) model. The K2P was considered as the most favourable model for small distance calculations^41^. Differences between intra- and inter-specific distances with four single barcodes were evaluated using pairwise distance matrix in MEGA X software. An ideal barcode could be determined with the presence of barcoding gap, that compared the intra- and inter- specific distance distribution for each barcode candidate with an interval distance of 0.05 in TaxonDNA with ‘pairwise summary function’^42^. In TaxonDNA, best match, best close match, and all species barcodes functions were intended to examine the accurate identification proportion of each barcode. The ‘Best match’ analyses determine the closet adjoining match for a known sequence. If the examined sequences were from the analogous species then the identification was considered correct whereas incorrect if the sequences belong to different species^29^.

### Phylogenetic analysis

The species discriminatory efficacy of each single and multi-locus barcode candidates was assessed with three tree-based method, which include the Neighbour-joining (NJ) tree, Maximum likelihood (ML) tree and Bayesian inference (BI) tree. The NJ methods of all markers were conducted using MEGA X^43,44^. The reliability of node was supported by bootstrap test of 1,000 pseudo-replicates with K2P distance parameter. For ML analysis, the phylogenetic trees were constructed in RAXMLGUI v1.3.1, a graphical front-end for RAXML^45^. The clade support was assessed using ML with thorough bootstrap analyses, run 10 times starting from random seeds under GTRGAMMA model and 1,000 non-parametric bootstrap values^46^. The species forming separate clusters in the tree with bootstrap support > 50% were considered to be distinct. The analysis of BI trees was conducted in MrBayes v3.2.7^47^. The best substitution models of each locus were selected according to Akaike information criterion (AIC) with jModeltest version 2.1.7^48^. The model suggested by jModeltest was GTR + I + G for all the tested barcode except GTR + G model for *mat*K. The two replicate runs of Markov chain Monte Carlo (MCMC) were run for 5,000,000 generations with four simultaneous chains (one cold and three hot chains), and trees were sampled at every 1000th generations. The adequate posterior probability (PP) distribution of samples were determined, when the split frequency of average standard deviation was lower than 0.01. Subsequently, the stationary was determined in Tracer v1.7.1^49^ and the first 25% trees were discarded as burn-in and a 50% majority-rule consensus tree was constructed and PP was considered as node support values. All the topologies of trees were visualized in FigTree v1.4.4. Percentages of species resolutions were calculated from the reconstructed tree in order to resolve the monophyletic nature of the clades. *Callicarpa americana*was used as an outgroup.

### Generation of QR code

The two-dimensional QR code consists of black modules with three squares on the corner of the code on white background and could involves 7,089 numeric, 4,296 alphanumeric characters, and 2,953 bytes of binary data^50^. In this study, the QR code image for the candidate barcode marker was generated by DNA QR Code Web Server^35^.

## Supplementary information

Supplementary Information

## Data Availability

We retrieved GenBank accessions of *ITS*2, *mat*K, *rbc*L and *ycf*1 for *Clerodendrum* sp. and details are included in Table S1 (Supporting Information). Submitted sequences of *Clerodendrum* species were included in Table S2.
