# Supplementary material for: DNA barcodes for delineating Clerodendrum species of North East India
Source: Sci Rep. 2020 Aug 10;10:13490. doi: 10.1038/s41598-020-70405-3 (PMC7417596; doi:10.1038/s41598-020-70405-3)
Supplement: Supplementary file 1 — Supplementary Information [file 41598_2020_70405_MOESM1_ESM.docx]

**DNA barcodes for delineating *Clerodendrum* species of North East India**

BARBI GOGOI^1,2^, S.B WANN^3^ and SP SAIKIA^1*^

^1^Medicinal Aromatic and Economic Plants Group, Biological Sciences & Technology Division (BSTD), CSIR-North East Institute of Science & Technology, Jorhat 785006, Assam, India

^2^Academy of Scientific and Innovative Research (AcSIR), Ghaziabad- 201002, India

^3^Biological science and Technology Group, Biological Sciences & Technology Division (BSTD), CSIR-North East Institute of Science & Technology, Jorhat 785006, Assam, India

*Corresponding author email: siddhartha@neist.res.in, spsaikia@gmail.com

Supplementary table:

Table S1: Sequences of *Clerodendrum* sp with four respective barcodes were retrieved from NCBI database

|  | Taxon | Voucher | Accession no. |
| --- | --- | --- | --- |
| *ITS*2 | *Clerodendrum bracteatum* | CB-AR 18S | KX228227 |
|  | *Clerodendrum bungei* | PS0853MT02 | FJ980369 |
|  | *Clerodendrum canescens* | SCBGP474_1 | KP092823 |
|  | *Clerodendrum canescens* | SCBGP474_2 | KP092824 |
|  | *Clerodendrum colebrookianum* | CC-AR-01 | KT728411 |
|  | *Clerodendrum colebrookianum* | MZU/BT/006 | KX079329 |
|  | *Clerodendrum cyrtophyllum* | Plant1 | KU745290 |
|  | *Clerodendrum cyrtophyllum* | Plant2 | KU745291 |
|  | *Clerodendrum cyrtophyllum* | Plant3 | KU745292 |
|  | *Clerodendrum cyrtophyllum* | Plant4 | KU745293 |
|  | *Clerodendrum cyrtophyllum* | HPS_a34 | KC896699 |
|  | *Clerodendrum cyrtophyllum* | SCBGP009_2 | KP092826 |
|  | *Clerodendrum heterophyllum* | CLHE_E.R._3of3 | KY700276 |
|  | *Clerodendrum heterophyllum* | CLHE_PL3_2of3 | KY700275 |
|  | *Clerodendrum heterophyllum* | CLHE_P.A_1of3 | KY700274 |
|  | *Clerodendrum indicum* | isolate CI-3 | KX828725 |
|  | *Clerodendrum indicum* | CIN-AS-13 | KT728413 |
|  | *Clerodendrum inerme* | AUFMS251 | KF848289 |
|  | *Clerodendrum infortunatum* | CV-AR-31 | KT728416 |
|  | *Clerodendrum japonicum* | CJ-AR-19 | KT728414 |
|  | *Clerodendrum japonicum* | PS0851MT01 | FJ980368 |
|  | *Clerodendrum japonicum* | J296 | KR531935 |
|  | *Clerodendrum japonicum* | J452 | KR531936 |
|  | *Clerodendrum japonicum* | J543 | KR531937 |
|  | *Clerodendrum japonicum* | SCBGP539_1 | KP092847 |
|  | *Clerodendrum japonicum* | SCBGP501_1 | KP092821 |
|  | *Clerodendrum japonicum* | SCBGP501_2 | KP092822 |
|  | *Clerodendrum philippinum* | CP-AR-25 | KT728415 |
|  | *Clerodendrum phlomidis* | FRLH-L/10/11/10 | KF199889 |
|  | *Clerodendrum thomsoniae* | CT-AR-37 | KT728417 |
|  | *Clerodendrum thomsoniae* | HPS_A04 | KC896701 |
|  | *Clerodendrum thomsoniae* | HPS_A03 | KC896700 |
|  | *Clerodendrum thomsoniae* | 0clethom | JN575348 |
|  | *Clerodendrum trichotomum* | P157 | JF980307 |
| *matK* | *Clerodendrum splendens* | Hosam00249 | KX783652 |
|  | *Clerodendrum laevifolium* | BT_0067702935 | KJ708870 |
|  | *Clerodendrum floribundum* | 129308661 | KM894594 |
|  | *Clerodendrum tomentosum* | 129308258 | KM894535 |
|  | *Clerodendrum cyrtophyllum* | YC0508MT01 | KJ888428 |
|  | *Clerodendrum cyrtophyllum* | YC0508MT02 | KJ939224 |
|  | *Clerodendrum cyrtophyllum* | YC0508MT03 | KJ939225 |
|  | *Clerodendrum cyrtophyllum* | SCBGP009_2 | KP093261 |
|  | *Clerodendrum cyrtophyllum* | SCBGP009_1 | KP093260 |
|  | *Clerodendrum japonicum* | J543 | KR530624 |
|  | *Clerodendrum japonicum* | J452 | KR530623 |
|  | *Clerodendrum japonicum* | J693 | KR530625 |
|  | *Clerodendrum japonicum* | J296 | KR530622 |
|  | *Clerodendrum japonicum* | SCBGP539_1 | KP094136 |
|  | *Clerodendrum japonicum* | SCBGP501_2 | KP094105 |
|  | *Clerodendrum japonicum* | SCBGP501_1 | KP094104 |
|  | *Clerodendrum trichotomum* | BOP010467 | KP089017 |
|  | *Clerodendrum philippinum* | SCBGP489_2 | KP094085 |
|  | *Clerodendrum philippinum* | SCBGP489_1 | KP094084 |
|  | *Clerodendrum canescens* | SCBGP474_2 | KP094059 |
|  | *Clerodendrum canescens* | SCBGP474_1 | KP094058 |
|  | *Clerodendrum fortunatum* | SCBGP118_2 | KP093457 |
|  | *Clerodendrum fortunatum* | SCBGP118_1 | KP093456 |
|  | *Clerodendrum glabrum* | OM768 | JF270694 |
|  | *Clerodendrum glabrum* | Abbott9161 | JX517832 |
|  | *Clerodendrum eriophyllum* | OM2759 | JX517512 |
| *rbcL* | *Clerodendrum infortunatum* | CIB | JQ724864 |
|  | *Clerodendrum infortunatum* | CIA | JQ724863 |
|  | *Clerodendrum quadriloculare* | CQ0614MT01 | JQ618470 |
|  | *Clerodendrum quadriloculare* | CQ0614MT02 | JQ618471 |
|  | *Clerodendrum quadriloculare* | M46 | KP208915 |
|  | *Clerodendrum splendens* | Hosam00249 | KX783849 |
|  | *Clerodendrum splendens* | CQ0613MT01 | JQ618469 |
|  | *Clerodendrum trichotomum* | 82 | HQ427186 |
|  | *Clerodendrum trichotomum* | BOP010467 | KP088539 |
|  | *Clerodendrum trichotomum* | 033816 | KJ688089 |
|  | *Clerodendrum trichotomum* | 158633 | KJ688088 |
|  | *Clerodendrum trichotomum* | 031673 | KJ688087 |
|  | *Clerodendrum trichotomum* | 246177 | KJ688086 |
|  | *Clerodendrum japonicum* | PS0851MT01 | GQ436521 |
|  | *Clerodendrum japonicum* | J543 | KR529030 |
|  | *Clerodendrum japonicum* | J452 | KR529029 |
|  | *Clerodendrum japonicum* | J693 | KR529031 |
|  | *Clerodendrum japonicum* | J296 | KR529028 |
|  | *Clerodendrum japonicum* | SCBGP539_1 | KP095101 |
|  | *Clerodendrum japonicum* | SCBGP501_2 | KP095059 |
|  | *Clerodendrum japonicum* | SCBGP501_1 | KP095058 |
|  | *Clerodendrum floribundum* | 129308661 | KM895703 |
|  | *Clerodendrum tomentosum* | 129308258 | KM895633 |
|  | *Clerodendrum cyrtophyllum* | YC0508MT02 | KJ939237 |
|  | *Clerodendrum cyrtophyllum* | YC0508MT01 | KJ939236 |
|  | *Clerodendrum cyrtophyllum* | YC0508MT03 | KJ939235 |
|  | *Clerodendrum cyrtophyllum* | SCBGP009_2 | KP094168 |
|  | *Clerodendrum cyrtophyllum* | SCBGP009_1 | KP094167 |
|  | *Clerodendrum cyrtophyllum* | A33384 | KJ688711 |
|  | *Clerodendrum cyrtophyllum* | 103492 | KJ688085 |
|  | *Clerodendrum cyrtophyllum* | 070175 | KJ688084 |
|  | *Clerodendrum cyrtophyllum* | 237047 | KJ688083 |
|  | *Clerodendrum cyrtophyllum* | 181019 | KJ688082 |
|  | *Clerodendrum cyrtophyllum* | CQ0605MT01 | JQ618457 |
|  | *Clerodendrum laevifolium* | BT_0067702935 | KJ594655 |
|  | *Clerodendrum philippinum* | SCBGP489_2 | KP095039 |
|  | *Clerodendrum philippinum* | SCBGP489_1 | KP095038 |
|  | *Clerodendrum philippinumvar. simplex* | CQ0607MT04 | JQ618462 |
|  | *Clerodendrum canescens* | SCBGP474_2 | KP095012 |
|  | *Clerodendrum canescens* | SCBGP474_1 | KP095011 |
|  | *Clerodendrum fortunatum* | SCBGP118_2 | KP094376 |
|  | *Clerodendrum fortunatum* | SCBGP118_1 | KP094375 |
|  | *Clerodendrum fortunatum* | CQ0602MT02 | JQ618450 |
|  | *Clerodendrum fortunatum* | CQ0602MT01 | JQ618449 |
|  | *Clerodendrum indicum* | CQ0612MT01 | JQ618468 |
|  | *Clerodendrum colebrookianum* | CQ0609MT01 | JQ618464 |
|  | *Clerodendrum bungei* | CQ0608MT01 | JQ618463 |
|  | *Clerodendrum wallichii* | CQ0604MT01 | JQ618455 |
|  | *Clerodendrum glabrum* | OM768 | JF265341 |
|  | *Clerodendrum glabrum* | Abbott9161 | JX572414 |
|  | *Clerodendrum eriophyllum* | OM2759 | JX572413 |
| *ycf*1 | *Clerodendrum trichotomum* | BOP010467 | KP088400 |

Table S2: Sequences submitted in NCBI database:

|  | Taxa | Isolate | Accession number in  NCBI |
| --- | --- | --- | --- |
| *ITS*2 | *Clerodendrum colebrookianum* | Ccol1 | MK132930 |
|  | *Clerodendrum colebrookianum* | Ccol2 | MK132931 |
|  | *Clerodendrum colebrookianum* | Ccol3 | MK132932 |
|  | *Clerodendrum colebrookianum* | Ccol4 | MK132933 |
|  | *Clerodendrum colebrookianum* | Ccol5 | MK132934 |
|  | *Clerodendrum colebrookianum* | Ccol6 | MK132935 |
|  | *Clerodendrum colebrookianum* | Ccol7 | MK132936 |
|  | *Clerodendrum colebrookianum* | Ccol8 | MK132937 |
|  | *Clerodendrum colebrookianum* | Ccol9 | MK132938 |
|  | *Clerodendrum colebrookianum* | Ccol10 | MK132939 |
|  | *Clerodendrum colebrookianum* | Ccol11 | MK132940 |
|  | *Clerodendrum colebrookianum* | Ccol12 | MK132941 |
|  | *Clerodendrum colebrookianum* | Ccol13 | MK132942 |
|  | *Clerodendrum colebrookianum* | Ccol14 | MK132943 |
|  | *Clerodendrum colebrookianum* | Ccol15 | MK132944 |
|  | *Clerodendrum colebrookianum* | Ccol16 | MK132945 |
|  | *Clerodendrum colebrookianum* | Ccol17 | MK132946 |
|  | *Clerodendrum infortunatum* | Cinf1 | MK132878 |
|  | *Clerodendrum infortunatum* | Cinf2 | MK132879 |
|  | *Clerodendrum infortunatum* | Cinf3 | MK132880 |
|  | *Clerodendrum infortunatum* | Cinf4 | MK132881 |
|  | *Clerodendrum infortunatum* | Cinf5 | MK132882 |
|  | *Clerodendrum infortunatum* | Cinf6 | MK132883 |
|  | *Clerodendrum infortunatum* | Cinf7 | MK132884 |
|  | *Clerodendrum infortunatum* | Cinf8 | MK132885 |
|  | *Clerodendrum infortunatum* | Cinf9 | MK132886 |
|  | *Clerodendrum infortunatum* | Cinf10 | MK132887 |
|  | *Clerodendrum infortunatum* | Cinf11 | MK132888 |
|  | *Clerodendrum infortunatum* | Cinf12 | MK132889 |
|  | *Clerodendrum infortunatum* | Cinf13 | MK132890 |
|  | *Clerodendrum infortunatum* | Cinf14 | MK132891 |
|  | *Clerodendrum philippinum* | Cphi1 | MK132919 |
|  | *Clerodendrum philippinum* | Cphi2 | MK132920 |
|  | *Clerodendrum philippinum* | Cphi3 | MK132921 |
|  | *Clerodendrum philippinum* | Cphi4 | MK132922 |
|  | *Clerodendrum philippinum* | Cphi5 | MK132923 |
|  | *Clerodendrum philippinum* | Cphi6 | MK132924 |
|  | *Clerodendrum philippinum* | Cphi7 | MK132925 |
|  | *Clerodendrum philippinum* | Cphi8 | MK132926 |
|  | *Clerodendrum philippinum* | Cphi9 | MK132927 |
|  | *Clerodendrum philippinum* | Cphi10 | MK132928 |
|  | *Clerodendrum philippinum* | Cphi11 | MK132929 |
|  | *Clerodendrum inerme* | Cine1 | MK132868 |
|  | *Clerodendrum inerme* | Cine2 | MK132869 |
|  | *Clerodendrum inerme* | Cine3 | MK132870 |
|  | *Clerodendrum inerme* | Cine4 | MK132871 |
|  | *Clerodendrum inerme* | Cine5 | MK132872 |
|  | *Clerodendrum inerme* | Cine6 | MK132873 |
|  | *Clerodendrum inerme* | Cine7 | MK132874 |
|  | *Clerodendrum inerme* | Cine8 | MK132875 |
|  | *Clerodendrum inerme* | Cine9 | MK132876 |
|  | *Clerodendrum inerme* | Cine10 | MK132877 |
|  | *Clerodendrum indicum* | Cind1 | MK132904 |
|  | *Clerodendrum indicum* | Cind2 | MK132905 |
|  | *Clerodendrum indicum* | Cind3 | MK132906 |
|  | *Clerodendrum indicum* | Cind4 | MK132907 |
|  | *Clerodendrum indicum* | Cind5 | MK132908 |
|  | *Clerodendrum indicum* | Cind6 | MK132909 |
|  | *Clerodendrum indicum* | Cind7 | MK132910 |
|  | *Clerodendrum indicum* | Cind8 | MK132911 |
|  | *Clerodendrum indicum* | Cind9 | MK132912 |
|  | *Clerodendrum indicum* | Cind10 | MK132913 |
|  | *Clerodendrum indicum* | Cind11 | MK132914 |
|  | *Clerodendrum indicum* | Cind12 | MK132915 |
|  | *Clerodendrum indicum* | Cind13 | MK132916 |
|  | *Clerodendrum indicum* | Cind14 | MK132917 |
|  | *Clerodendrum indicum* | Cind15 | MK132918 |
|  | *Clerodendrum serratum* | Cser1 | MK132951 |
|  | *Clerodendrum serratum* | Cser2 | MK132952 |
|  | *Clerodendrum serratum* | Cser3 | MK132953 |
|  | *Clerodendrum serratum* | Cser4 | MK132954 |
|  | *Clerodendrum serratum* | Cser5 | MK132955 |
|  | *Clerodendrum japonicum* | Cjap1 | MK132892 |
|  | *Clerodendrum japonicum* | Cjap2 | MK132893 |
|  | *Clerodendrum japonicum* | Cjap3 | MK132894 |
|  | *Clerodendrum japonicum* | Cjap4 | MK132895 |
|  | *Clerodendrum japonicum* | Cjap5 | MK132896 |
|  | *Clerodendrum japonicum* | Cjap6 | MK132897 |
|  | *Clerodendrum japonicum* | Cjap7 | MK132898 |
|  | *Clerodendrum paniculatum* | Cpan1 | MK132899 |
|  | *Clerodendrum paniculatum* | Cpan2 | MK132900 |
|  | *Clerodendrum paniculatum* | Cpan3 | MK132901 |
|  | *Clerodendrum paniculatum* | Cpan4 | MK132902 |
|  | *Clerodendrum paniculatum* | Cpan5 | MK132903 |
|  | *Clerodendrum thomsoniae* | Cthom1 | MK132947 |
|  | *Clerodendrum thomsoniae* | Cthom2 | MK132948 |
|  | *Clerodendrum thomsoniae* | Cthom6 | MK132949 |
|  | *Clerodendrum thomsoniae* | Cthom7 | MK132950 |
| *matK* | *Clerodendrum colebrookianum* | Ccol1 | MK521980 |
|  | *Clerodendrum colebrookianum* | Ccol2 | MK5464484 |
|  | *Clerodendrum colebrookianum* | Ccol3 | MK521981 |
|  | *Clerodendrum colebrookianum* | Ccol4 | MK521982 |
|  | *Clerodendrum colebrookianum* | Ccol5 | MK521983 |
|  | *Clerodendrum colebrookianum* | Ccol6 | MK521984 |
|  | *Clerodendrum colebrookianum* | Ccol7 | MK521985 |
|  | *Clerodendrum colebrookianum* | Ccol8 | MK521986 |
|  | *Clerodendrum colebrookianum* | Ccol9 | MK521987 |
|  | *Clerodendrum colebrookianum* | Ccol10 | MK521988 |
|  | *Clerodendrum colebrookianum* | Ccol11 | MK551750 |
|  | *Clerodendrum colebrookianum* | Ccol12 | MK551751 |
|  | *Clerodendrum colebrookianum* | Ccol13 | MK551752 |
|  | *Clerodendrum colebrookianum* | Ccol14 | MK551753 |
|  | *Clerodendrum colebrookianum* | Ccol15 | MK551754 |
|  | *Clerodendrum colebrookianum* | Ccol16 | MK551755 |
|  | *Clerodendrum colebrookianum* | Ccol17 | MK551756 |
|  | *Clerodendrum colebrookianum* | Ccol18 | MK551757 |
|  | *Clerodendrum infortunatum* | Cinf1 | MK551758 |
|  | *Clerodendrum infortunatum* | Cinf2 | MK551759 |
|  | *Clerodendrum infortunatum* | Cinf3 | MK551760 |
|  | *Clerodendrum infortunatum* | Cinf4 | MK551761 |
|  | *Clerodendrum infortunatum* | Cinf5 | MK551762 |
|  | *Clerodendrum infortunatum* | Cinf7 | MK551763 |
|  | *Clerodendrum infortunatum* | Cinf8 | MK551764 |
|  | *Clerodendrum infortunatum* | Cinf9 | MK551765 |
|  | *Clerodendrum infortunatum* | Cinf10 | MK551766 |
|  | *Clerodendrum infortunatum* | Cinf11 | MK551767 |
|  | *Clerodendrum infortunatum* | Cinf12 | MK551768 |
|  | *Clerodendrum infortunatum* | Cinf13 | MK551769 |
|  | *Clerodendrum infortunatum* | Cinf14 | MK551770 |
|  | *Clerodendrum philippinum* | Cphi1 | MK551771 |
|  | *Clerodendrum philippinum* | Cphi2 | MK551772 |
|  | *Clerodendrum philippinum* | Cphi3 | MK551773 |
|  | *Clerodendrum philippinum* | Cphi4 | MK551774 |
|  | *Clerodendrum philippinum* | Cphi5 | MK551775 |
|  | *Clerodendrum philippinum* | Cphi6 | MK551776 |
|  | *Clerodendrum philippinum* | Cphi7 | MK551777 |
|  | *Clerodendrum philippinum* | Cphi8 | MK551778 |
|  | *Clerodendrum philippinum* | Cphi9 | MK551779 |
|  | *Clerodendrum philippinum* | Cphi10 | MK551780 |
|  | *Clerodendrum inerme* | Cine1 | MK551781 |
|  | *Clerodendrum inerme* | Cine2 | MK551782 |
|  | *Clerodendrum inerme* | Cine3 | MK551783 |
|  | *Clerodendrum inerme* | Cine4 | MK551784 |
|  | *Clerodendrum inerme* | Cine5 | MK551785 |
|  | *Clerodendrum inerme* | Cine6 | MK551786 |
|  | *Clerodendrum inerme* | Cine7 | MK551787 |
|  | *Clerodendrum inerme* | Cine8 | MK551788 |
|  | *Clerodendrum inerme* | Cine9 | MK551789 |
|  | *Clerodendrum inerme* | Cine10 | MK551790 |
|  | *Clerodendrum indicum* | Cind1 | MK551791 |
|  | *Clerodendrum indicum* | Cind2 | MK551792 |
|  | *Clerodendrum indicum* | Cind3 | MK551793 |
|  | *Clerodendrum indicum* | Cind4 | MK551794 |
|  | *Clerodendrum indicum* | Cind5 | MK551795 |
|  | *Clerodendrum indicum* | Cind7 | MK551796 |
|  | *Clerodendrum indicum* | Cind8 | MK551797 |
|  | *Clerodendrum indicum* | Cind9 | MK551798 |
|  | *Clerodendrum indicum* | Cind10 | MK551799 |
|  | *Clerodendrum indicum* | Cind11 | MK551800 |
|  | *Clerodendrum indicum* | Cind12 | MK551801 |
|  | *Clerodendrum indicum* | Cind14 | MK551802 |
|  | *Clerodendrum indicum* | Cind15 | MK551803 |
|  | *Clerodendrum serratum* | Cser1 | MK551804 |
|  | *Clerodendrum serratum* | Cser2 | MK551805 |
|  | *Clerodendrum serratum* | Cser3 | MK551806 |
|  | *Clerodendrum serratum* | Cser4 | MK551807 |
|  | *Clerodendrum serratum* | Cser5 | MK551808 |
|  | *Clerodendrum serratum* | Cser6 | MK551809 |
|  | *Clerodendrum japonicum* | Cjap1 | MK551810 |
|  | *Clerodendrum japonicum* | Cjap2 | MK551811 |
|  | *Clerodendrum japonicum* | Cjap3 | MK551812 |
|  | *Clerodendrum japonicum* | Cjap4 | MK551813 |
|  | *Clerodendrum japonicum* | Cjap5 | MK551814 |
|  | *Clerodendrum japonicum* | Cjap6 | MK551815 |
|  | *Clerodendrum japonicum* | Cjap7 | MK551816 |
|  | *Clerodendrum japonicum* | Cjap8 | MK551817 |
|  | *Clerodendrum paniculatum* | Cpan1 | MK551818 |
|  | *Clerodendrum paniculatum* | Cpan2 | MK551819 |
|  | *Clerodendrum paniculatum* | Cpan3 | MK551820 |
|  | *Clerodendrum paniculatum* | Cpan4 | MK551821 |
|  | *Clerodendrum paniculatum* | Cpan5 | MK551822 |
|  | *Clerodendrum thomsoniae* | Ctho1 | MK551823 |
|  | *Clerodendrum thomsoniae* | Ctho2 | MK5521827 |
|  | *Clerodendrum thomsoniae* | Ctho3 | MK5521828 |
|  | *Clerodendrum thomsoniae* | Ctho4 | MK5521829 |
|  | *Clerodendrum thomsoniae* | Ctho5 | MK551824 |
|  | *Clerodendrum thomsoniae* | Ctho6 | MK551825 |
|  | *Clerodendrum thomsoniae* | Ctho7 | MK551826 |
| *rbcL* | *Clerodendrum colebrookianum* | Ccol1 | MK226730 |
|  | *Clerodendrum colebrookianum* | Ccol2 | MK241951 |
|  | *Clerodendrum colebrookianum* | Ccol3 | MK241952 |
|  | *Clerodendrum colebrookianum* | Ccol4 | MK226731 |
|  | *Clerodendrum colebrookianum* | Ccol5 | MK241953 |
|  | *Clerodendrum colebrookianum* | Ccol6 | MK241954 |
|  | *Clerodendrum colebrookianum* | Ccol7 | MK241955 |
|  | *Clerodendrum colebrookianum* | Ccol8 | MK241960 |
|  | *Clerodendrum colebrookianum* | Ccol9 | MK241961 |
|  | *Clerodendrum colebrookianum* | Ccol10 | MK241964 |
|  | *Clerodendrum colebrookianum* | Ccol11 | MK241956 |
|  | *Clerodendrum colebrookianum* | Ccol12 | MK241957 |
|  | *Clerodendrum colebrookianum* | Ccol13 | MK241962 |
|  | *Clerodendrum colebrookianum* | Ccol14 | MK241958 |
|  | *Clerodendrum colebrookianum* | Ccol15 | MK241965 |
|  | *Clerodendrum colebrookianum* | Ccol16 | MK241966 |
|  | *Clerodendrum colebrookianum* | Ccol17 | MK241963 |
|  | *Clerodendrum colebrookianum* | Ccol18 | MK241959 |
|  | *Clerodendrum infortunatum* | Cinf1 | MK331741 |
|  | *Clerodendrum infortunatum* | Cinf2 | MK331742 |
|  | *Clerodendrum infortunatum* | Cinf3 | MK331743 |
|  | *Clerodendrum infortunatum* | Cinf4 | MK331744 |
|  | *Clerodendrum infortunatum* | Cinf5 | MK331745 |
|  | *Clerodendrum infortunatum* | Cinf6 | MK331754 |
|  | *Clerodendrum infortunatum* | Cinf7 | MK331746 |
|  | *Clerodendrum infortunatum* | Cinf8 | MK331747 |
|  | *Clerodendrum infortunatum* | Cinf9 | MK331748 |
|  | *Clerodendrum infortunatum* | Cinf10 | MK331749 |
|  | *Clerodendrum infortunatum* | Cinf11 | MK331750 |
|  | *Clerodendrum infortunatum* | Cinf12 | MK331751 |
|  | *Clerodendrum infortunatum* | Cinf13 | MK331752 |
|  | *Clerodendrum infortunatum* | Cinf14 | MK331753 |
|  | *Clerodendrum philippinum* | Cphi1 | MK377303 |
|  | *Clerodendrum philippinum* | Cphi2 | MK331773 |
|  | *Clerodendrum philippinum* | Cphi3 | MK331774 |
|  | *Clerodendrum philippinum* | Cphi4 | MK331775 |
|  | *Clerodendrum philippinum* | Cphi5 | MK331776 |
|  | *Clerodendrum philippinum* | Cphi6 | MK331777 |
|  | *Clerodendrum philippinum* | Cphi7 | MK331778 |
|  | *Clerodendrum philippinum* | Cphi8 | MK331779 |
|  | *Clerodendrum philippinum* | Cphi9 | MK331780 |
|  | *Clerodendrum philippinum* | Cphi10 | MK331781 |
|  | *Clerodendrum inerme* | Cine1 | MK331755 |
|  | *Clerodendrum inerme* | Cine2 | MK331756 |
|  | *Clerodendrum inerme* | Cine3 | MK331782 |
|  | *Clerodendrum inerme* | Cine4 | MK331783 |
|  | *Clerodendrum inerme* | Cine5 | MK331784 |
|  | *Clerodendrum inerme* | Cine6 | MK331757 |
|  | *Clerodendrum inerme* | Cine7 | MK331758 |
|  | *Clerodendrum inerme* | Cine8 | MK331759 |
|  | *Clerodendrum inerme* | Cine9 | MK331760 |
|  | *Clerodendrum inerme* | Cine10 | MK331761 |
|  | *Clerodendrum indicum* | Cind1 | MK331785 |
|  | *Clerodendrum indicum* | Cind2 | MK331786 |
|  | *Clerodendrum indicum* | Cind3 | MK331787 |
|  | *Clerodendrum indicum* | Cind4 | MK331788 |
|  | *Clerodendrum indicum* | Cind5 | MK331789 |
|  | *Clerodendrum indicum* | Cind7 | MK331790 |
|  | *Clerodendrum indicum* | Cind8 | MK331791 |
|  | *Clerodendrum indicum* | Cind9 | MK331792 |
|  | *Clerodendrum indicum* | Cind10 | MK331793 |
|  | *Clerodendrum indicum* | Cind11 | MK331794 |
|  | *Clerodendrum indicum* | Cind12 | MK377304 |
|  | *Clerodendrum indicum* | Cind13 | MK331795 |
|  | *Clerodendrum indicum* | Cind14 | MK331796 |
|  | *Clerodendrum serratum* | Cser3 | MK331797 |
|  | *Clerodendrum serratum* | Cser6 | MK331798 |
|  | *Clerodendrum japonicum* | Cjap1 | MK331763 |
|  | *Clerodendrum japonicum* | Cjap2 | MK331764 |
|  | *Clerodendrum japonicum* | Cjap3 | MK331765 |
|  | *Clerodendrum japonicum* | Cjap4 | MK331799 |
|  | *Clerodendrum japonicum* | Cjap5 | MK331766 |
|  | *Clerodendrum japonicum* | Cjap6 | MK331800 |
|  | *Clerodendrum japonicum* | Cjap7 | MK331801 |
|  | *Clerodendrum paniculatum* | Cpan1 | MK331767 |
|  | *Clerodendrum paniculatum* | Cpan2 | MK331768 |
|  | *Clerodendrum paniculatum* | Cpan3 | MK331769 |
|  | *Clerodendrum paniculatum* | Cpan4 | MK331770 |
|  | *Clerodendrum paniculatum* | Cpan5 | MK331771 |
|  | *Clerodendrum thomsoniae* | Ctho1 | MK331802 |
|  | *Clerodendrum thomsoniae* | Ctho2 | MK331772 |
|  | *Clerodendrum thomsoniae* | Ctho3 | MK331803 |
|  | *Clerodendrum thomsoniae* | Ctho5 | MK331804 |
|  | *Clerodendrum thomsoniae* | Ctho6 | MK331805 |
|  | *Clerodendrum thomsoniae* | Ctho7 | MK331806 |
|  | *Clerodendrum colebrookianum* | Ccol1 | MK602221 |
|  | *Clerodendrum colebrookianum* | Ccol2 | MK602222 |
|  | *Clerodendrum colebrookianum* | Ccol3 | MK602223 |
|  | *Clerodendrum colebrookianum* | Ccol4 | MK602224 |
|  | *Clerodendrum colebrookianum* | Ccol5 | MK602225 |
|  | *Clerodendrum colebrookianum* | Ccol6 | MK602226 |
|  | *Clerodendrum colebrookianum* | Ccol7 | MK602227 |
|  | *Clerodendrum colebrookianum* | Ccol8 | MK602228 |
|  | *Clerodendrum colebrookianum* | Ccol9 | MK602229 |
|  | *Clerodendrum colebrookianum* | Ccol10 | MK602230 |
|  | *Clerodendrum colebrookianum* | Ccol11 | MK602231 |
|  | *Clerodendrum colebrookianum* | Ccol12 | MK602232 |
|  | *Clerodendrum colebrookianum* | Ccol13 | MK602233 |
|  | *Clerodendrum colebrookianum* | Ccol14 | MK602234 |
|  | *Clerodendrum colebrookianum* | Ccol15 | MK602235 |
|  | *Clerodendrum colebrookianum* | Ccol16 | MK602236 |
|  | *Clerodendrum colebrookianum* | Ccol17 | MK602237 |
|  | *Clerodendrum colebrookianum* | Ccol18 | MK602238 |
|  | *Clerodendrum infortunatum* | Cinf1 | MK574576 |
|  | *Clerodendrum infortunatum* | Cinf2 | MK602239 |
|  | *Clerodendrum infortunatum* | Cinf3 | MK602240 |
|  | *Clerodendrum infortunatum* | Cinf4 | MK602241 |
|  | *Clerodendrum infortunatum* | Cinf5 | MK602242 |
|  | *Clerodendrum infortunatum* | Cinf6 | MK602243 |
|  | *Clerodendrum infortunatum* | Cinf8 | MK602244 |
|  | *Clerodendrum infortunatum* | Cinf9 | MK602245 |
|  | *Clerodendrum infortunatum* | Cinf10 | MK602246 |
|  | *Clerodendrum infortunatum* | Cinf11 | MK602247 |
|  | *Clerodendrum infortunatum* | Cinf12 | MK602248 |
|  | *Clerodendrum infortunatum* | Cinf13 | MK602249 |
|  | *Clerodendrum infortunatum* | Cinf14 | MK602250 |
|  | *Clerodendrum philippinum* | Cphi2 | MK602251 |
|  | *Clerodendrum philippinum* | Cphi3 | MK602252 |
|  | *Clerodendrum philippinum* | Cphi4 | MK602253 |
|  | *Clerodendrum philippinum* | Cphi5 | MK602254 |
|  | *Clerodendrum philippinum* | Cphi6 | MK602255 |
|  | *Clerodendrum philippinum* | Cphi7 | MK602256 |
|  | *Clerodendrum philippinum* | Cphi8 | MK602257 |
|  | *Clerodendrum philippinum* | Cphi9 | MK602258 |
|  | *Clerodendrum philippinum* | Cphi10 | MK602259 |
|  | *Clerodendrum philippinum* | Cphi11 | MK602260 |
|  | *Clerodendrum inerme* | Cine1 | MK602261 |
|  | *Clerodendrum inerme* | Cine2 | MK602262 |
|  | *Clerodendrum inerme* | Cine3 | MK602263 |
|  | *Clerodendrum inerme* | Cine4 | MK602264 |
|  | *Clerodendrum inerme* | Cine5 | MK602265 |
|  | *Clerodendrum inerme* | Cine6 | MK602266 |
|  | *Clerodendrum inerme* | Cine7 | MK602267 |
|  | *Clerodendrum inerme* | Cine8 | MK602268 |
|  | *Clerodendrum inerme* | Cine9 | MK602269 |
|  | *Clerodendrum inerme* | Cine10 | MK602270 |
|  | *Clerodendrum indicum* | Cind1 | MK602271 |
|  | *Clerodendrum indicum* | Cind2 | MK602272 |
|  | *Clerodendrum indicum* | Cind3 | MK602273 |
|  | *Clerodendrum indicum* | Cind4 | MK602274 |
|  | *Clerodendrum indicum* | Cind5 | MK602275 |
|  | *Clerodendrum indicum* | Cind6 | MK602276 |
|  | *Clerodendrum indicum* | Cind7 | MK602277 |
|  | *Clerodendrum indicum* | Cind8 | MK602278 |
|  | *Clerodendrum indicum* | Cind9 | MK602279 |
|  | *Clerodendrum indicum* | Cind10 | MK602280 |
|  | *Clerodendrum indicum* | Cind11 | MK602281 |
|  | *Clerodendrum indicum* | Cind12 | MK602282 |
|  | *Clerodendrum indicum* | Cind13 | MK602283 |
|  | *Clerodendrum indicum* | Cind14 | MK602284 |
|  | *Clerodendrum indicum* | Cind15 | MK602285 |
|  | *Clerodendrum serratum* | Cser1 | MK602286 |
|  | *Clerodendrum serratum* | Cser2 | MK602287 |
|  | *Clerodendrum serratum* | Cser3 | MK602288 |
|  | *Clerodendrum serratum* | Cser4 | MK602289 |
|  | *Clerodendrum serratum* | Cser5 | MK602290 |
|  | *Clerodendrum serratum* | Cser6 | MK602291 |
|  | *Clerodendrum japonicum* | Cjap1 | MK602292 |
|  | *Clerodendrum japonicum* | Cjap2 | MK602293 |
|  | *Clerodendrum japonicum* | Cjap3 | MK602294 |
|  | *Clerodendrum japonicum* | Cjap4 | MK602295 |
|  | *Clerodendrum japonicum* | Cjap5 | MK602296 |
|  | *Clerodendrum japonicum* | Cjap6 | MK602297 |
|  | *Clerodendrum japonicum* | Cjap7 | MK602298 |
|  | *Clerodendrum japonicum* | Cjap8 | MK602299 |
|  | *Clerodendrum paniculatum* | Cpan1 | MK602300 |
|  | *Clerodendrum paniculatum* | Cpan2 | MK602301 |
|  | *Clerodendrum paniculatum* | Cpan3 | MK602302 |
|  | *Clerodendrum paniculatum* | Cpan4 | MK602303 |
|  | *Clerodendrum paniculatum* | Cpan5 | MK602304 |
|  | *Clerodendrum thomsoniae* | Ctho1 | MK602305 |
|  | *Clerodendrum thomsoniae* | Ctho2 | MK602306 |
|  | *Clerodendrum thomsoniae* | Ctho5 | MK602307 |
|  | *Clerodendrum thomsoniae* | Ctho6 | MK602308 |
| *ycf*1 | *Clerodendrum colebrookianum* | Ccol1 | MK602221 |
|  | *Clerodendrum colebrookianum* | Ccol2 | MK602222 |
|  | *Clerodendrum colebrookianum* | Ccol3 | MK602223 |
|  | *Clerodendrum colebrookianum* | Ccol4 | MK602224 |
|  | *Clerodendrum colebrookianum* | Ccol5 | MK602225 |
|  | *Clerodendrum colebrookianum* | Ccol6 | MK602226 |
|  | *Clerodendrum colebrookianum* | Ccol7 | MK602227 |
|  | *Clerodendrum colebrookianum* | Ccol8 | MK602228 |
|  | *Clerodendrum colebrookianum* | Ccol9 | MK602229 |
|  | *Clerodendrum colebrookianum* | Ccol10 | MK602230 |
|  | *Clerodendrum colebrookianum* | Ccol11 | MK602231 |
|  | *Clerodendrum colebrookianum* | Ccol12 | MK602232 |
|  | *Clerodendrum colebrookianum* | Ccol13 | MK602233 |
|  | *Clerodendrum colebrookianum* | Ccol14 | MK602234 |
|  | *Clerodendrum colebrookianum* | Ccol15 | MK602235 |
|  | *Clerodendrum colebrookianum* | Ccol16 | MK602236 |
|  | *Clerodendrum colebrookianum* | Ccol17 | MK602237 |
|  | *Clerodendrum colebrookianum* | Ccol18 | MK602238 |
|  | *Clerodendrum infortunatum* | Cinf1 | MK574576 |
|  | *Clerodendrum infortunatum* | Cinf2 | MK602239 |
|  | *Clerodendrum infortunatum* | Cinf3 | MK602240 |
|  | *Clerodendrum infortunatum* | Cinf4 | MK602241 |
|  | *Clerodendrum infortunatum* | Cinf5 | MK602242 |
|  | *Clerodendrum infortunatum* | Cinf6 | MK602243 |
|  | *Clerodendrum infortunatum* | Cinf8 | MK602244 |
|  | *Clerodendrum infortunatum* | Cinf9 | MK602245 |
|  | *Clerodendrum infortunatum* | Cinf10 | MK602246 |
|  | *Clerodendrum infortunatum* | Cinf11 | MK602247 |
|  | *Clerodendrum infortunatum* | Cinf12 | MK602248 |
|  | *Clerodendrum infortunatum* | Cinf13 | MK602249 |
|  | *Clerodendrum infortunatum* | Cinf14 | MK602250 |
|  | *Clerodendrum philippinum* | Cphi2 | MK602251 |
|  | *Clerodendrum philippinum* | Cphi3 | MK602252 |
|  | *Clerodendrum philippinum* | Cphi4 | MK602253 |
|  | *Clerodendrum philippinum* | Cphi5 | MK602254 |
|  | *Clerodendrum philippinum* | Cphi6 | MK602255 |
|  | *Clerodendrum philippinum* | Cphi7 | MK602256 |
|  | *Clerodendrum philippinum* | Cphi8 | MK602257 |
|  | *Clerodendrum philippinum* | Cphi9 | MK602258 |
|  | *Clerodendrum philippinum* | Cphi10 | MK602259 |
|  | *Clerodendrum philippinum* | Cphi11 | MK602260 |
|  | *Clerodendrum inerme* | Cine1 | MK602261 |
|  | *Clerodendrum inerme* | Cine2 | MK602262 |
|  | *Clerodendrum inerme* | Cine3 | MK602263 |
|  | *Clerodendrum inerme* | Cine4 | MK602264 |
|  | *Clerodendrum inerme* | Cine5 | MK602265 |
|  | *Clerodendrum inerme* | Cine6 | MK602266 |
|  | *Clerodendrum inerme* | Cine7 | MK602267 |
|  | *Clerodendrum inerme* | Cine8 | MK602268 |
|  | *Clerodendrum inerme* | Cine9 | MK602269 |
|  | *Clerodendrum inerme* | Cine10 | MK602270 |
|  | *Clerodendrum indicum* | Cind1 | MK602271 |
|  | *Clerodendrum indicum* | Cind2 | MK602272 |
|  | *Clerodendrum indicum* | Cind3 | MK602273 |
|  | *Clerodendrum indicum* | Cind4 | MK602274 |
|  | *Clerodendrum indicum* | Cind5 | MK602275 |
|  | *Clerodendrum indicum* | Cind6 | MK602276 |
|  | *Clerodendrum indicum* | Cind7 | MK602277 |
|  | *Clerodendrum indicum* | Cind8 | MK602278 |
|  | *Clerodendrum indicum* | Cind9 | MK602279 |
|  | *Clerodendrum indicum* | Cind10 | MK602280 |
|  | *Clerodendrum indicum* | Cind11 | MK602281 |
|  | *Clerodendrum indicum* | Cind12 | MK602282 |
|  | *Clerodendrum indicum* | Cind13 | MK602283 |
|  | *Clerodendrum indicum* | Cind14 | MK602284 |
|  | *Clerodendrum indicum* | Cind15 | MK602285 |
|  | *Clerodendrum serratum* | Cser1 | MK602286 |
|  | *Clerodendrum serratum* | Cser2 | MK602287 |
|  | *Clerodendrum serratum* | Cser3 | MK602288 |
|  | *Clerodendrum serratum* | Cser4 | MK602289 |
|  | *Clerodendrum serratum* | Cser5 | MK602290 |
|  | *Clerodendrum serratum* | Cser6 | MK602291 |
|  | *Clerodendrum japonicum* | Cjap1 | MK602292 |
|  | *Clerodendrum japonicum* | Cjap2 | MK602293 |
|  | *Clerodendrum japonicum* | Cjap3 | MK602294 |
|  | *Clerodendrum japonicum* | Cjap4 | MK602295 |
|  | *Clerodendrum japonicum* | Cjap5 | MK602296 |
|  | *Clerodendrum japonicum* | Cjap6 | MK602297 |
|  | *Clerodendrum japonicum* | Cjap7 | MK602298 |
|  | *Clerodendrum japonicum* | Cjap8 | MK602299 |
|  | *Clerodendrum paniculatum* | Cpan1 | MK602300 |
|  | *Clerodendrum paniculatum* | Cpan2 | MK602301 |
|  | *Clerodendrum paniculatum* | Cpan3 | MK602302 |
|  | *Clerodendrum paniculatum* | Cpan4 | MK602303 |
|  | *Clerodendrum paniculatum* | Cpan5 | MK602304 |
|  | *Clerodendrum thomsoniae* | Ctho1 | MK602305 |
|  | *Clerodendrum thomsoniae* | Ctho2 | MK602306 |
|  | *Clerodendrum thomsoniae* | Ctho5 | MK602307 |
|  | *Clerodendrum thomsoniae* | Ctho6 | MK602308 |
